# Supplementary material for: Meta-Analysis and Gene Set Enrichment Relative to ER Status Reveal Elevated Activity of MYC and E2F in the “Basal” Breast Cancer Subgroup
Source: PLoS One. 2009 Mar 9;4(3):e4710. doi: 10.1371/journal.pone.0004710 (PMC2650420; doi:10.1371/journal.pone.0004710)
Supplement: Dataset S1 — GSEA results from first screen. The file contains a zipped archive of the HTML results of the second GSEA screen. Reports for enriched or depleted datasets may be accessed from the “index.html” link within the my_analysis.GseaPreranked.1219975950625.rpt.zip ZIP archive. Enrichment plots are available for each of the 50 most enriched and depleted gene sets. (13.32 MB ZIP) [file pone.0004710.s001.zip › my_analysis.GseaPreranked.1219975950625.rpt.zip!//REN_E2F1_TARGETS.html]

Details for gene set REN\_E2F1\_TARGETS[GSEA]

|  || Dataset | zSco.ERneg.to.ERpos\_collapsed |
| Phenotype | NoPhenotypeAvailable |
| Upregulated in class | na\_pos |
| GeneSet | REN\_E2F1\_TARGETS |
| Enrichment Score (ES) | 0.66222984 |
| Normalized Enrichment Score (NES) | 2.2597432 |
| Nominal p-value | 0.0 |
| FDR q-value | 0.0 |
| FWER p-Value | 0.0 |
Table: GSEA Results Summary

  

Fig 1: Enrichment plot: REN\_E2F1\_TARGETS      
 Profile of the Running ES Score & Positions of GeneSet Members on the Rank Ordered List

  

| PROBE | GENE SYMBOL | GENE\_TITLE | RANK IN GENE LIST | RANK METRIC SCORE | RUNNING ES | CORE ENRICHMENT || 1 | E2F3 |  |  | 6 | 7.369 | 0.0755 | Yes |
| 2 | MCM5 |  |  | 31 | 6.533 | 0.1409 | Yes |
| 3 | RAD54L |  |  | 146 | 5.602 | 0.1901 | Yes |
| 4 | RFC3 |  |  | 164 | 5.493 | 0.2454 | Yes |
| 5 | CDC25A |  |  | 281 | 4.993 | 0.2881 | Yes |
| 6 | RFC4 |  |  | 370 | 4.741 | 0.3303 | Yes |
| 7 | MCM3 |  |  | 656 | 4.019 | 0.3502 | Yes |
| 8 | NCL |  |  | 678 | 3.977 | 0.3896 | Yes |
| 9 | FEN1 |  |  | 754 | 3.838 | 0.4235 | Yes |
| 10 | RFC2 |  |  | 762 | 3.821 | 0.4623 | Yes |
| 11 | MSH2 |  |  | 819 | 3.748 | 0.4967 | Yes |
| 12 | PRIM2A |  |  | 941 | 3.564 | 0.5243 | Yes |
| 13 | MYC |  |  | 1025 | 3.447 | 0.5536 | Yes |
| 14 | UNG |  |  | 1070 | 3.390 | 0.5852 | Yes |
| 15 | PRKDC |  |  | 1234 | 3.204 | 0.6059 | Yes |
| 16 | CDC2 |  |  | 1259 | 3.182 | 0.6369 | Yes |
| 17 | PCNA |  |  | 1346 | 3.087 | 0.6622 | Yes |
| 18 | CBX5 |  |  | 2621 | 2.133 | 0.5883 | No |
| 19 | POLA2 |  |  | 2649 | 2.117 | 0.6081 | No |
| 20 | UMPS |  |  | 3124 | 1.877 | 0.5917 | No |
| 21 | MTHFD1 |  |  | 3258 | 1.814 | 0.6004 | No |
| 22 | TOP2A |  |  | 3735 | 1.610 | 0.5812 | No |
| 23 | KIAA0101 |  |  | 4770 | 1.230 | 0.5160 | No |
| 24 | RRM1 |  |  | 4872 | 1.197 | 0.5207 | No |
| 25 | CALR |  |  | 5146 | 1.099 | 0.5115 | No |
| 26 | STAM |  |  | 6423 | 0.674 | 0.4224 | No |
| 27 | SFRS2 |  |  | 6677 | 0.597 | 0.4095 | No |
| 28 | DUT |  |  | 6839 | 0.539 | 0.4029 | No |
| 29 | SMARCA3 |  |  | 7380 | 0.352 | 0.3659 | No |
| 30 | RB1 |  |  | 8000 | 0.105 | 0.3204 | No |
| 31 | PRPSAP1 |  |  | 8951 | -0.285 | 0.2518 | No |
| 32 | ID3 |  |  | 8993 | -0.308 | 0.2519 | No |
| 33 | SLC3A2 |  |  | 9152 | -0.383 | 0.2440 | No |
| 34 | CALM2 |  |  | 9592 | -0.613 | 0.2172 | No |
| 35 | RPA3 |  |  | 11357 | -1.892 | 0.1039 | No |
| 36 | MLH1 |  |  | 12924 | -4.246 | 0.0298 | No |
Table: GSEA details [plain text format]

  

Fig 2: REN\_E2F1\_TARGETS: Random ES distribution      
 Gene set null distribution of ES for **REN\_E2F1\_TARGETS**
